# Bautin bifurcations in a forest-grassland ecosystem with human-environment interactions

**DOI:** 10.1038/s41598-019-39296-x

**Published:** 2019-02-25

**Authors:** Lucia Russo, Konstantinos Spiliotis, Francesco Giannino, Stefano Mazzoleni, Constantinos Siettos

**Affiliations:** 1 Consiglio Nazionale delle Ricerche, Istituto di Ricerche sulla Combustione, Naples, 80125 Italy; 2 Universitá degli Studi di Napoli Federico II, Dipartimento di Agraria, Laboratorio di Ecologia Applicata e Sistemi Dinamici, Portici (NA), 80055 Italy; 3 Universitá degli Studi di Napoli Federico II, Dipartimento di Matematica e Applicazioni “Renato Caccioppoli”, Naples, 80126 Italy

## Abstract

Ecosystems may be characterized by a complex dynamical behaviour where external disturbances and/or internal perturbations may trigger sudden/irreversible changes, called catastrophic shifts. Simple mathematical models in the form of ordinary and/or partial differential equations have been proposed to approximate in a qualitatively manner the observed complex phenomena, where catastrophic shifts are determined by bifurcation points. In this work, we show that in ecosystems, gradual/smooth changes may be transformed in sudden/catastrophic shifts as a consequence of codimension-2 bifurcations. We stress the importance of using the full arsenal of numerical bifurcation theory to systematically identify and characterize criticalities in ecological models in the 2D parameter space. For our demonstrations, we revisit the analysis of a simple model of a forest-grassland mosaic ecosystem constructing the 2D bifurcation diagram with respect to the impact of human influence and that of natural causes. Our numerical analysis reveals that this simple model is able to approximate both abrupt (catastrophic) and smooth transitions as the system undergoes Bautin bifurcations.

## Introduction

The systematic modeling, analysis and forecasting of the complex dynamical behaviour of ecosystems in response to their ongoing changes constitutes one of the major challenges of nowadays. It is now well recognized, that many ecosystems are characterized by a complex dynamical behaviour^1,2^. Thus, external disturbances such as volcanic eruptions, hurricanes and lightning-caused wildfires, and internal state perturbations such as biodiversity loss and forest harvesting may trigger sudden/irreversible changes called catastrophic shifts. From a nonlinear dynamics perspective, a sudden shift becomes more probable if the system is close to a criticality, i.e. close to a bifurcation point which marks the onset of phase transitions. For example, it has been shown, that the extinction of logistically growing populations^3^ and that of the vegetation of shallow lakes^4^ occur often through transcritical bifurcations. Abrupt shifts to the vegetation biomass in valleys^1,5^, bogs^6^ and ocean-climate changes^7,8^ are proven to be triggered by fold bifurcations. Phase transitions in ocean-climate systems may also occur through supercritical Andronov-Hopf points accompanied by homoclinic bifurcations^7^. A subcritical pitchfork bifurcation has been suggested as the mechanism of the Atlantic thermohaline circulation^9,10^, while a subcritical Andronov-Hopf bifurcation has been suggested as the mechanism of sudden large-amplitude stable oscillations in forest/grassland ecosystems under human influence^11,12^.

Hence, the vast majority of the studies highlight the importance of bifurcation theory to analyse, understand and ultimately forecast tipping points that mark the onset of regime shifts^13–23^. However, despite the powerful arsenal of numerical bifurcation theory, many studies still use simple temporal simulations and/or simple linear numerical analysis to explore the systems behaviour and to identify criticalities. However, simple temporal simulations and/or linear stability analysis may not reveal the whole picture. Such “simple” analysis may miss important information (e.g regarding the characterization of nonlinear phenomena in the parameter space) which in turn can lower our ability to correctly identify and characterize critical points and therefore the mechanisms that pertain to the birth of the emergent phenomena.

**Figure 1:**
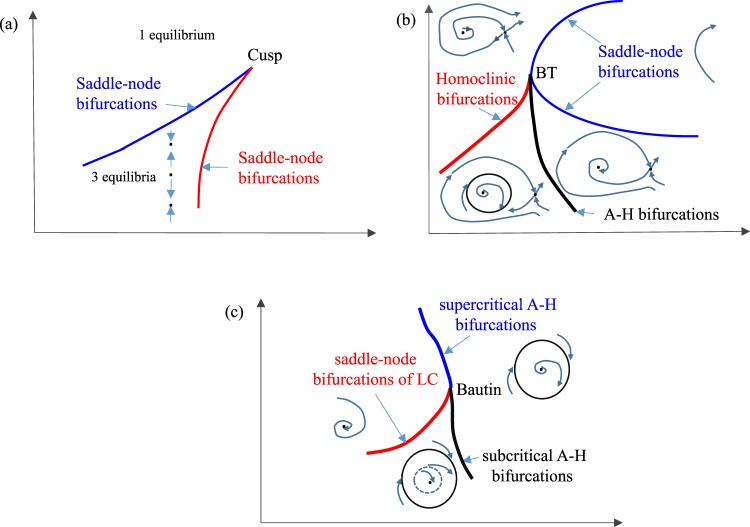
Schematic of characteristic 2D bifurcations. (**a**) Cusp, (**b**) Bogdanov-Takens (BD), (**c**) Bautin (generalized Andronov-Hopf). Schematics of phase-portraits in the different regimes are also shown.

In this paper, we stress the importance of using the full arsenal of numerical bifurcation analysis and particularly that of codimension-2 bifurcation analysis to systematically identify and characterize criticalities in ecological models. The codimension of a bifurcation reflects the number of parameters that are necessary for the bifurcation to occur. Thus, a codimension-2 bifurcation analysis implies the detection and trace of bifurcations in the 2D parametric space. These codimension-2 bifurcations include (a schematic of such bifurcations is shown in Fig. 1)^24–26^ cusp points where two saddle-node bifurcation branches meet tangentially (Fig. 1(a)) and set limits between multiple and single equilibria, Bogdanov-Takens points which mark the coincide of saddle-node bifurcation (turning) points with Andronov-Hopf and saddle homoclinic bifurcations (Fig. 1(b)) setting the separatrices of stable and unstable oscillations and homoclinc orbits, Bautin (called also generalized Andronov-Hopf) points which mark the border between branches of subcritical, supercritical Andronov-Hopf bifurcations and turning points of limit cycles (Fig. 1(c)), thus setting the separatrices betwen stable and unstable oscillations. Other codimension-2 bifurcations include fold-Andronov-Hopf bifurcations lying at the intersection of curves of saddle-node bifurcations and Andronov-Hopf bifurcation curves, cusp-Andronov-Hopf points referring to the coincide of Andronov-Hopf with cusp points and double Andronov-Hopf points, where for example, two Andronov-Hopf bifurcation curves intersect transversally^24–26^.

At this point, we should note that from an ecological point of view, simple low-dimensional models that have been used for the analysis of ecological systems can be only regarded as caricatures of the real-phenomena observed. Thus, low-dimensional models have been criticized as being (at best) illustrative. Furthermore, it has been shown that the dynamics of such models can change when adding further degrees of freedom^27^. This has been recognized early on for the Daisyworld model^28,29^, a simple planetary model describing the long-term effects of the coupling between life and its environment. Recent studies, aiming to predict tipping points, make considerably efforts to address the issues of modelling robustness^30^.

Here, in order to convey our concerns, we focus on the example of forest-grassland mosaic ecosystems which have been shown to undergo abrupt/catastrophic shifts^11^. In these systems, two ecological states (forest and grassland) compete for the same source such as soil, sunlight and space. While the literature is rich of model studies where bistability is theoretically predicted in an isolated system, much less is known about the dynamics of a forest/grassland ecosystem in the presence of human interactions. Frequently, the external disturbances in ecosystems are caused by human activities, and the understanding of how disturbances influence their dynamics is very important in order to prevent and control ecological disasters that may manifest as a shift to undesired stable states^11,12,31–35^.

Here, we complete the analysis of the forest-grassland ecosystem model dynamics proposed in^11^, by performing a codimension-2 bifurcation analysis. Compared with the temporal simulations and linear analysis presented in^11^ and for the same values of parameters, our analysis reveals unseen complex behaviour. We show that this simple model can be used to qualitatively approximate a wide range of transitions observed in nature. In particular, our analysis unfolds both smooth and catastrophic shifts including regimes of reversible transitions of oscillatory patterns emerging from supercritical Andronov-Hopf bifurcations, as well as smooth transitions of oscillatory patterns followed by irreversible abrupt jumps to stable equilibria due to fold points of limit cycles.

## Results

**Figure 2:**
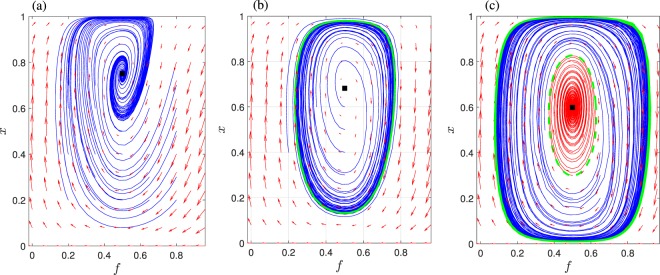
Phase portraits for different (*ν*, *k*) values; here h=1/2 (weak interaction), however qualitatively same behaviour is observed for *h*=2 (strong human interaction). Regime (**a**) with a unique stable equilibrium (*ν* = 0.9, *k* = 20), (**b**) where an unstable equilibrium coexists with a stable limit cycle (*ν* = 0.5, *k* = 10), (**c**) where a stable equilibrium coexists with an unstable and a stable limit cycle (*ν* = 0.2, *k* = 6). Arrows indicate the direction of the flow field, whereas solid lines correspond to trajectories for different initial conditions. Equilibrium points are marked by black squares; stable limit cycles are shown as thick solid green lines; unstable limit cycles are shown as thick dashed green lines (as opposed to the thin lines that show trajectories).

The model under analysis consists of two ordinary differential equations describing the dynamics of a mosaic ecosystem with two states, namely forest and grassland in which an exogenous input, that of the human interaction is introduced as a feedback (see Methods and Materials). Despite its simplicity, the dynamics of the model addressed in Innes *et al*.^11^, exhibit a rich behaviour. As already discussed^11^, the system presents multiplicity of stable steady states and oscillatory regimes in a wide range of the parameter space. In particular, for *k* ∈ [0, 50], *ν* ∈ [0, 3] and for a wide range of the parameter *h*  spanning weak and strong human interactions (the other parameters are set to *b* = 11, *c* = 1, *s* = 10), three types of phase portraits may by observed (see Fig. 2 for a qualtitative overview): (a) a stable equilibrium as unique regime (Fig. 2(a)); (b) a stable limit cycle surrounding an unstable equilibrium (Fig. 2(b)), and, (c) coexistence of a stable and an unstable limit cycle surrounding a stable equilibrium (Fig. 2(c)).

In the first case, all the trajectories converge to the equilibrium, in the second case to the stable limit cycle, whereas in the last case, the basins of attraction of the two attractors are separated by the unstable limit cycle. Starting with initial conditions within the region delimited by the unstable limit cycle, the trajectories end up to the stable equilibrium, otherwise converge to the stable limit cycle. Thus, a weak perturbation of the stable equilibrium that is surrounded by the unstable limit cycle does not change the asymptotic behavior of the system; instead a strong perturbation (crossing the basin of attraction of the stable equilibrium) will lead the system to a different regime characterized by stable oscillations. As the perturbation is applied to the vector state, this transition can be seen as a catastrophic shift. To delimit in the parameter space the regions where different dynamical behaviour (different phase portraits) exist, we constructed the 2D bifurcation diagram in the (*k*, *ν*) parameter plane, by performing a codimension-2 numerical continuation of the codimension-1 bifurcations. Our motivation was to draw the map of the dynamical behaviour in the two-dimensional parameter space (*k*, *ν*) (which was constructed in Innes *et al*.^11^ by temporal simulations (see Fig. 2c^11^)) using the tools of codimension-2 numerical bifurcation analysis^25,26^. For the bifurcation analysis, we have set *h* = 2.

**Figure 3:**
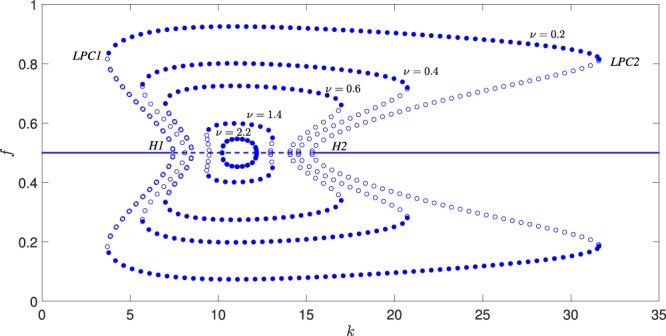
1D Bifurcation diagrams considering *k* as the bifurcation parameter. For values of *ν* less than a critical value, subcritical Andronov-Hopf bifurcations [(*ν* = 0.2, *k* = 7.42, *k* = 15.31), (*ν* = 0.4, *k* = 8.09, *k* = 14.54), (*ν* = 0.6, *k* = 8.47, *k* = 14.06), (*ν* = 1.4, *k* = 9.48, *k* = 12.96)] give rise to catastrophic transitions between equilibria and oscillating solutions. For larger values of *ν*, the Andronov-Hopf bifurcations become supercritical (*ν* = 2.2, *k* = 10.22, *k* = 12.17) marking the onset of smooth transitions. Solid lines refer to stable equilibria, whereas the dashed line between the two Andronov-Hopf points denote unstable equilibria. Limit cycles are indicated with circles; filled (empty) circles correspond to stable (unstable) limit cycles; *H* indicates Andronov-Hopf bifurcations and *LPC* indicate turning points of limit cycles.

We first computed the 1D bifurcation diagrams for different values of *ν* considering *k* as the bifurcation parameter. The 1D-bifurcation diagrams allowed us to analyze the origin of the oscillating regimes, to detect in a systematic way the multiplicity parameter regions as well as to compute the critical parameter values where transitions occur; these tasks cannot be resolved in a rigorous way by temporal simulations and/or linear analysis alone. The results of our 1D bifurcation analysis with respect to *k* for different values of *ν* are illustrated in Fig. 3.

**Figure 4:**
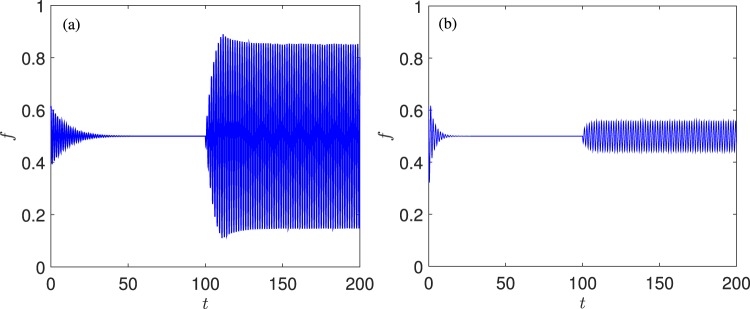
Shift from the stable equilibrium to stable oscillations as a consequence of the perturbation of the vector state and/or the value of the bifurcation parameter  *k* (see also Fig. 3). (**a**) Catastrophic shift due to a perturbation to the vector state and/or a change of the value of the bifurcation parameter for *ν* = 0.2, *k* = 30; this catastrophic shift is caused due to the subcritical Andronov-Hopf bifurcation *H2*. (**b**) Smooth shift for *ν* = 2, due to a supercritical Andronov-Hopf bifurcation (here changing *k* = 13 to * k* = 11).  The perturbations were imposed at *t* = 100.

For small values of *ν* and until *ν* = 1.768, the dynamics are characterized by two subcritical Andronov-Hopf bifurcation points marked with *H*1, *H*2. From *H*1, a branch of unstable limit cycles arises gaining back stability through a turning point bifurcation of limit cycles (*LPC*1). The stable limit cycles disappear on a second turning point of limit cycles (*LPC*2). Thus, stable oscillations exist in the range [*LPC*1; *LPC*2]. A branch of unstable limit cycles connects *LPC*2 with the second subcritical Andronov-Hopf bifurcation, *H*2. It is clear, that for these values of parameters, there are two ranges of bistability characterized by the coexistence of a stable equilibrium and a stable limit cycle, namely [*LPC*1; *H*1] and [*H*2; *LPC*2]. In these bistability regions, the phase portrait is the one shown in Fig. 2(c), thus implying the possibility of an abrupt transition (catastrophic shift) as a consequence of a perturbation of the vector state which cross the basin boundary of the two coexisting stable regimes. On the other hand, two catastrophic shifts may be observed as a consequence of parameter perturbation, which are both due to the presence of the two subcritical Andronov-Hopf bifurcations. The first one may occur if the system is in a stable equilibrium which is close to one of the two Andronov-Hopf bifurcations. In this range of *ν* values, a (small) perturbation of the parameter *k* leads to an abrupt change from the equilibrium (characterized by a fixed percent of forest) to an oscillating behaviour with large amplitudes. A catastrophic shift due to a perturbation to the vector state and/or a change in the bifurcation parameter *k* is illustrated in Fig. 4(a). On the contrary, when increasing the value of *ν* (to larger values than 1.768), the Andronov-Hopf bifurcations (first *H*2 and then *H*1) become supercritical marking the passage to a completely different scenario, where the shift from one regime to the other is smooth and thus not catastrophic (see Fig. 4(b)). It should be also noted that when the two Andronov-Hopf bifurcations become supercritical, the two *LPC* points, which mark two catastrophic bifurcations disappear. Indeed, if the system is in a periodic regime close to the two *LPC* points, a small perturbation of the parameter may lead the system suddenly to an equilibrium regime. Thus, the passage of *H* points from subcritical to supercritical, and vice versa, marks the passage from a situation where more than one catastrophic shifts may occur, to a condition in which the transition from the equilibrium to the oscillatory regime (and vice versa) is smooth.

**Figure 5:**
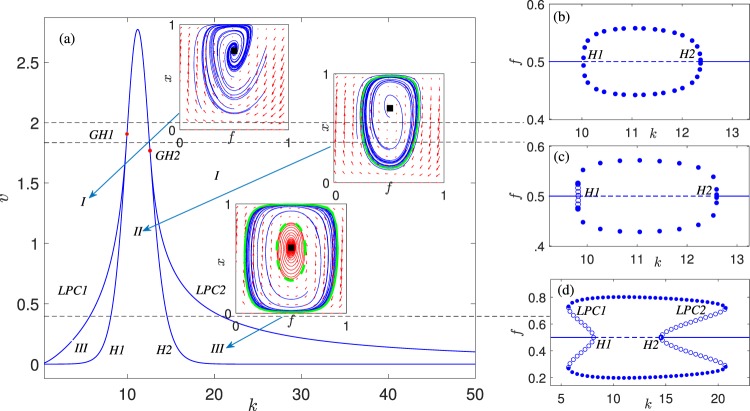
(**a**) Codimension-2 bifurcation diagram in the (*k*, *v*) parameter space. 1D bifurcations diagrams with respect to *k* for (**b**) *v* = 2; in this case there are two supercritical Andronov-Hopf points at *k* = 10.03 and *k* = 12.37, (**c**) *v* = 1.82; in this case there is one subcritical at *k* = 9.82 and one supercritical Andronov-Hopf bifurcation at *k* = 12.59, (**d**) *v* = 0.4; in this case there are two subcritical Andronov-Hopf points at *k* = 8.09 and *k* = 14.54. Solid lines refer to stable equilibrium solutions, whereas dashed lines refer to unstable ones. Stable (unstable) limit cycles are indicated with filled (empty) circles.

From a nonlinear dynamics point of view, this passage is marked by codimension-2 bifurcations which can be detected systematically by computing the 2D bifurcation diagram with respect to the parameters *ν* and *k*. Thus, after computing *H*1, *H*2, *LPC*1 and *LPC*2 for a specific couple (*k*, *ν*), we performed a 2D-parameter continuation in order to trace the locus of all the codimension-1 bifurcations in the (*k*, *ν*) parameter plane. The results of the codimension-2 numerical analysis are shown in Fig. 5. The central bell-like curve in Fig. 5(a) (marked with *H*1, *H*2) corresponds to the locus of Andronov-Hopf points and the two side curves (marked with *LPC*1 and *LPC*2) correspond to the limit point bifurcations of limit cycles. When the branches of the *LPC* points (which correspond to the collapse of stable-unstable limit cycles) cross the locus of Andronov-Hopf points, a generalized Andronov-Hopf bifurcation (it is also called a Bautin bifurcation) occurs at the points (*f*, *x*, *k*, *ν*) = (1/2,0.722, 9.946, 1.906) (*GH*1) and (*f*, *x*, *k*, *ν*) = (1/2, 0.669, 12.59, 1.768) (*GH*2). In these codimension-2 bifurcations, both the conditions for Andronov-Hopf and limit point bifurcations are met. The parameter plane is partitioned from the bifurcation curves into regions with different dynamics. In particular, the regions in Fig. 5(a) denoted as I, II and III, refer to the phase-plane dynamics reported in Fig. 2(a–c), respectively. As *ν* increases, both the range of existence of the limit cycles and the bistability range decrease. The different 1D bifurcation diagrams (regarding *k* as bifurcation parameter for different characteristic values of *ν*) are reported in Fig. 5(b,c,d) and they reveal different scenarios of dynamic phase transitions in the mosaic environment.

It is clear that these scenarios are classified by the two Bautin bifurcations (*GH*1, *GH*2), where both the Andronov-Hopf and *LPC* point critical conditions are met. For values of *ν* larger than those of the *GH*1 Bautin bifurcation, the 1D bifurcation diagram with respect to *k* exhibits two supercritical Andronov-Hopf bifurcations Fig. 5(b). Thus, around these bifurcations, small perturbations in *k* result to smooth shifts; for increasing values of *k*, just after the first Andronov-Hopf bifurcation *H*1, oscillatory patterns of small amplitude emerge. For larger values of *k* past *H*1, the amplitude of the oscillating patterns increases up to a point after which it gradually decreases until the bifurcation point *H*2 at which the oscillating patterns disappear. Thus, for relatively large values of the natural conversion rate *ν* (*f* → *g*) and independently of the *k* parameter, the system undergoes smooth changes, making the system less influenced by the fire incidence.

Within a narrow range of *ν* values, between the values of the two Bautin bifurcations *GH*1 and *GH*2, a different scenario emerges. As an example, Fig. 5(c) depicts the 1D bifurcation diagram with respect to *k* for *ν* = 1.82. In this case, the Andronov-Hopf bifurcation that emerges at *k* = 9.82 is subcritical, thus giving rise to sudden transitions; small perturbations of the *k* past the first critical point *H*1 give rise to high amplitude oscillations. Instead at *k* = 12.59 the Andronov-Hopf point (*H*2) is supercritical, thus marking the onset of smooth transitions between equilibria and oscillating patterns.

Finally, for *ν* values smaller than the corresponding critical values of the two Bautin bifurcations, *GH*1 and *GH*2, both Andronov-Hopf bifurcations are subcritical (Fig. 5(d)), thus marking the onset of abrupt transitions between equilibria and oscillating solutions.

Bautin bifurcations occur also in several other parameter regimes. For example, for *b* = 11, *c* = 0.4, *h* = 2, *s* = 10, the Bautin bifurcations occur at (*f*, *x*, *k*, *v*) = (0.5, 0.588, 9.94, 0.76) and at (*f*, *x*, *k*, *v*) = (0.5, 0.567, 12.592, 0.707), and for *b* = 4, *c* = 1, *h* = 2, *s* = 10, the Bautin bifurcations occur at (*f*, *x*, *k*, *v*) = (0.5, 0.569, 3.274, 0.719) and at (*f*, *x*, *k*, *v*) = (0.5, 0.522, 6.024, 0.62).

## Discussion

Sudden (catastrophic) shifts in ecosystems can be triggered either by a state perturbation, in which the solution may jump to a different stable state because of the crossing of the boundaries of its basin of attraction, or by a (small) change of the values of a parameter. Such catastrophic regime shifts take place around turning points (of equilibria or of limit cycles), subcritical pitchfork, subcritical Andronov-Hopf bifurcations and global bifurcations such as homoclinic bifurcations where a stable limit cycle hits a saddle equilibrium and disappears. Another scenario can also happen. Instead of a sudden catastrophic change, a smooth non-catastrophic transition to another phase is possible for example through a transcritical bifurcation^36^, a supercritical pitchfork or a supercritical Andronov-Hopf bifurcation that marks the onset of sustained stable oscillations. A different scenario may involve both abrupt and smooth changes: states emanating from a bifurcation resulting to smooth phase transitions may loose abruptly their resilience close to a catastrophic criticality.

The above codimension-1 bifurcations can give a systematic insight when a single parameter is varied. However, it is often the case that one would like to track critical points when also another parameter is varied. For example, it is known that the El-Niño Southern Oscillation resonances are strongly affected by two parameters, the mean and the annual variation of the ocean-atmosphere coupling^37^. In such cases, codimension-2 bifurcations arise. Examples of systems that fall in the above complexity include the transitions between El Niño and La Niña states due to the presence/disappearance of multiple equilibria^18,38^, the oceanic thermohaline circulation over the ages exhibiting stable and unstable oscillations and homoclinic orbits^39,40^ and as shown here, both smooth and catastrophic transitions between equilibria and oscillating patterns.

A very recent paper has shown how such bifurcation analysis and the detection of codimension-2 bifurcations may enlighten the mechanisms pertaining to the complex behaviour in even very simple mathematical models of forest-grassland mosaic ecosystems^41^.

All in all, while temporal simulations and linear stability analysis are the first tasks one can employ to study the dynamics of an ecological model, they are often inadequate to systematically investigate and reveal the mechanisms that give rise to the observed complex behaviour in the parameter space. For example (especially for medium to large-scale models of ordinary and/or partial differential equations), simple temporal simulations and linear stability analysis are inadequate to detect off-equilibrium unstable solutions and in turn their stable manifolds which constitute the separatrices of the basis of attractions of different regimes. Thus, in order to better understand and characterize rigorously and in detail the emerged nonlinear behaviour one should resort to the arsenal of codimension-1 and codimension-2 numerical bifurcation analysis. Here, to convey the above necessity, we performed a complete 2D bifurcation analysis of a mosaic ecological model addressed in Innes *et al*.^11^. By doing so, we show that a region containing a single stable interior equilibrium accompanied by an unstable limit cycle which was anticipated from temporal simulations^11^ does not exist; as we show an unstable limit cycle does not exist in this region, instead only a stable equilibrium exists. Importantly, we found a range in the 2D parameter space where the reported^11^ subcritical Andronov-Hopf bifurcations, become supercritical through a Bautin bifurcation, thus marking the passage to a completely different scenario (reported for the first time) where the shift from one regime to the other is smooth and thus not catastrophic. A physical explanation that can be given for the two qualitatively different transitions is the following. For small values of the natural conversion rate from forest to grassland the system becomes very sensitive to the impact of fire. Thus, the fire may lead the system to sharp changes due to the two subcrtitical Andronov-Hopf bifurcations. As the conversion rate increases, within the range of Bautin bifurcations, the grassland is reproduced fast, but this reproduction is interrupted (or prevented) by a faster response of the community to reforestation. In this way, due to the action-reaction mechanism, the sharp transition can be reduced, thus leading to smoothly (oscillating) shifts.

To this end, we should comment that for the particular values of the parameters used to construct the 2D bifurcation diagram depicted in Fig. 5 the Bautin bifurcations occur around *v* ≈ 2/*year* (as the unit of time in the model of Innes *et al*.^11^ is one year). This is a natural rate corresponding to a clearance of a forest to grassland every six months, which is rather unrealistic. However, the selection of one year as unit of time should not be regarded as restrictive. If for example, one assumes as unit of time one decade, then such a value of the parameter *v*, where Bautin bifurcations occur can be plausible. Thus taking the decade as unit of time, values of the model parameters such as the ones selected for our illustrations may be realistic depending on the environmental conditions. For example, the parameter *c* reflects the maximum rate of forest recruitment when the mediation of fire is negligible. This value depends on many environmental factors, which allow a wide range of variability. Apart from this fact, as we showed, Bautin bifurcations arise also for smaller values of the parameter *v* for different sets of values of the other parameters, thus the model may predict “realistic” scenarios of Bautin bifurcations even for the one-year time-unit scale.

## Methods and Materials

### The Dynamical Model

Here, we describe briefly the model proposed by Innes *et al*.^11^ on the dynamics of a simple mosaic ecosystem with two states, namely forest and grassland. Let *f* and *g* be the proportion of forest and grassland in the ecosystem, respectively. The evolution of the forest is approximated by the following equation:

1 $$\frac{df}{dt}=w(f)gf-\nu f$$

where *ν* express the transition rate from forest to grassland due to natural processes and *w*(*f*) is the rate of transition from grassland to forest mainly induced due to fire: in the forest-grassland mosaic environment, the fire destroys completely small plants but not mature trees, i.e. favours *g* → *f* conversion. Larger values of the forest density *f* results to larger values of *w*(*f*) (leading to effective regeneration of forest) and consequently a dense forest resists better to fire than a sparse forested grassland. The transition from low to high regeneration rates is almost sharp, with *w*(*f*) reading:

2 $$w(f)=\frac{c}{1+{e}^{-k\frac{f}{1-f}+b}}$$

where *b*, *c* and *k* are parameters and *k* is related to the fast activation of *w*. Assuming no human intervention and the conservation relation *f* + *g* = 1, the evolution of forest density is described by a 1D dynamical system which takes the form:

3 $$\frac{df}{dt}=w(f)f\mathrm{(1}-f)-\nu f$$

The human influence is modelled as follows (for more details see Innes *et al*.^11^).

The population is divided into two categories, those that prefer forest and those that prefer grassland. Let *x* be the proportion of the people that prefer forest; then 1 − *x* is the ratio of people that prefer grassland. Each individual can alter his/her opinion according to a mimetic (majority) rule. Thus, the interaction of the same category of individuals does not effect opinion, whereas an individual that prefers forest (grassland) changes his/her opinion with a social learning rate *s* if he/she interacts with an individual that prefers grassland (forest)^11^. Furthermore, the rate of change depends on the perceived value of forest *u*_*f*_(*f*) and that of grassland *u*_*g*_(*f*) which depend on the relative abundance of grassland and forest. For example, *u*_*g*_(*f*) is small when the forest is rare and the grassland is abundant and large when the grassland is rare and the forest is abundant. All in all, the perceived value of forest versus that of grassland is modelled in the general case as

4 $$u(f)=r{g}^{m}-q{f}^{n}$$

In the above relation, *m* and *n* control the shape of the function, *r* is the gain for the perceived value of forest and *q* is the gain for the perceived value of grassland. Here, for our illustrations we have set *m* = *n* = 1 and *r* = *q* = 1, thus obtaining *u*(*f*) = (1 − *f*) − *f* = 1 − 2*f*.

According to the above, the dynamics of the population *x* is described by the equation:

5 $$\frac{dx}{dt}=sx\mathrm{(1}-x)u(f)$$

The influence of human behaviour on forest/grassland dynamics can be expressed by adding a positive/negative feedback. In our system, this feedback is represented by the term *J*(*x*) = *h*(1 − 2*x*), where *h* is the potential magnitude of human influence on the ecosystem. Human interaction adds positive or negative feedback in the forest density according to the social preferences using the following rule: if the majority prefers forest (i.e. *x* > 1/2) the society will enhance forest recreation (i.e −*J*(*x*) > 0) by reforestation; instead if social opinion favours grassland then the human impact leads to deforestation (i.e −*J*(*x*) < 0). Thus the coupled model becomes:

6 $$\{\begin{array}{l}\frac{df}{dt}=w(f)(1-f)f-\nu f-J(x)\\ \frac{dx}{dt}=sx(1-x)u(f)\end{array}$$

### Numerical bifurcation analysis software tools

Numerical bifurcation analysis theory provides an arsenal of algorithms and software packages, such as AUTO^42,43^, MATCONT^44,45^, XPPAUT^46,47^, CONTENT^48–50^ and COCO^51,52^ for tasks such as the continuation of stable, unstable steady states and limit cycles, and the continuation of codimension-2 bifurcations. Here, for our computations we used MATCONT setting the absolute and relative tolerances of Newton iterations equal to 1E-6.
